# Immutable Morality

**Published:** 1866-11

**Authors:** J. R. W. Sloane


### IMMUTABLE MORALITY.

BY J. R. W. SLOANE.

"It is impossible for God to lie." Why? Because it is directly contrary to his nature. The foundation of moral obligation is to be sought, not in the *will*, but in the *nature* of God. A thing is right or wrong, not merely because God commands or forbids it, but the right and the wrong of the thing are the reason of its injunction or inhibition. It follows, that the laws of moral obligation are as unchangeable as God—as immutable as the pillars of his throne. The principles of his moral government are the same from age to age eternally. If, in former periods, God did not pour out his judgment upon men, while in the indulgence of practices offensive to his holiness and in violation of his law, but manifested his goodness by sending them rain from heaven, and fruitful seasons, filling their hearts with joy and gladness; this was because, as the great Apostle of the Gentiles informs the Athenians, when he stood on Mars' Hill, "The times of this ignorance God winked at, but now commandeth all men everywhere to repent."

Unchanged by time, they cannot be altered or annulled by circumstances; these may "alter cases." But, whatever this trite aphorism may mean, it cannot apply to those cases into which enter principles of moral obligation. To practice idolatry, to dishonor parents, to violate the Sabbath, to murder, commit adultery, steal, lie, or blaspheme, are sins at all times, and under all circumstances, which no one can practice and be free from guilt, no matter what the incitement, temptation, or provocation to their commission may be: these may soften the judgments of men, palliate the guilt before earthly tribunals, may be passed before the scrutinies of a merciful God, but cannot abrogate the law, or remove the solid foundations upon which an immutable morality rests.

By the moral law of God, man is bound as an individual, a member of the family, the church, and the commonwealth, wherever great interests are discussed or transacted—in the editorial chair, at the bar, in the pulpit, on the platform, in the legislative halls of the nation, on its benches of justice, in its cabinet counsels, at the head of its armies—in short, wherever he does or can act as a moral or accountable being; for their violation, he cannot plead the will of a superior, whether that of a single individual or a multitude, but must say, "The Lord our God will we obey, and him only will we serve." No pressure of circumstances, or sanction of law, by way of reward or penalty, can be plead—"Whether it is better to obey God or man, judge ye." As well may yon twinkling star expect to break away from the law that binds it to its orbit, and wander at will throughout the Universe, as for man to suppose that he can in any way divest himself of his moral accountability, and place himself outside of the sphere of moral obligation.

These laws, also—a truth which we are too prone to forget—bind nationalities as well as individuals. Nations, it is true, have no souls; but those who compose them, have. Politicians may divest themselves of conscience—the *soul* remains, and they will be held to a rigid account as individuals for the manner in which they have discharged their duty, and wielded this influence and power, which, for wise and great purposes and ends, God has committed to them; and in addition to this, if nations will affront his Throne, and defy his power, as nations they must suffer the penalty of his violated and outraged law.

If for them there is no *future*, there are tremendous temporal punishments, as is plainly written upon almost every page of his Word and his Providence. In his hand is a cup of red wine, which he commends to their guilty lips, and thunderbolts of wasting and consuming vengeance, which, as Horace says, by their crimes, they will not permit him to lay aside—a truth confirmed by the ruins of mighty empires which were, but are not; by the desolations of lands once populous, powerful, and prosperous, which for sins of those who dwelt therein, have been turned into barrenness.

France revoked the Edict of Nantes, shed the blood of the saints like water, upon the bloody day of St. Bartholomew, memorable in the annals of the Church and the world; and but a few years had come and gone, until she had blood to drink, for she was worthy—wasted by wars, and weeping for the best and bravest of her children, whose bleaching bones whitened every plain of Europe, from the Seine to the Volga.

Polona fell, not, it is true, unwept, nor yet, we fear, without a crime, but by injustice the most foul and atrocious that stains the pages of modern history. On the bloody battle fields of the Crimea, and before the frowning battlements of Sebastopol, a righteous God inflicted disgrace and punishment upon the proudest of her spoilers.

How often, too, does the punishment tread upon the very heels of crime. The sorrowful wail of crushed and down-trodden Hungary has scarcely died away. This very morning, the news that war is inevitable is flashed along the wires. The mightiest nations of Europe are about to "let slip the dogs of war" upon perfidious Austria, on the world's past and future battle fields—the plains of Italy. She must drink the cup of trembling to the very dregs, which, through long ages of inflicted oppression and wrong, she has been filling, and which has been made finally to overflow, by the crimes of the present reigning dynasty, the perjured house of Hapsburg. In these and other similar and equally remarkable Providences, which we might trace, did time permit, shall we not hear the voice of God himself from the high Imperial Throne of the Universe, saying, "Be wise now, therefore, O ye kings, be instructed, ye judges of the earth—kiss the Son, lest he be angry, and ye perish from the way when his wrath is kindled but a little: till heaven and earth pass away, one jot or one tittle shall in no wise pass from the law till all be fulfilled."

May the words which form the caption of this article arrest the attention of every one whose eye may chance to fall upon them, as they did that of the writer long years ago, when turning the pages of a work more learned, perhaps, and profound, but in all probability not more useful, than the "*Fireside Monthly*." May they take it with them as a motto through life; and may our rulers be warned by the example of other nations in the past and the present, and remember, that for nations there is only a future of desolation and ruin, unless they practice upon the principles of an "Immutable Morality," and incorporate them into the constitution and free administration of government.

